# Phosphorylation of a splice variant of collapsin response mediator protein 2 in the nucleus of tumour cells links cyclin dependent kinase-5 to oncogenesis

**DOI:** 10.1186/s12885-015-1691-1

**Published:** 2015-11-10

**Authors:** Nicola J. Grant, Philip J. Coates, Yvonne L. Woods, Susan E. Bray, Nicholas A. Morrice, C. James Hastie, Douglas J. Lamont, Francis A. Carey, Calum Sutherland

**Affiliations:** 1 Division of Cardiovascular and Diabetes Medicine, University of Dundee, Ninewells Medical School, DD1 9SY, Dundee, UK; 2 Division of Cancer, University of Dundee, Dundee, UK; 3 Department of Pathology, Ninewells Hospital, NHS Tayside, Dundee, UK; 4 Beatson Cancer Institute, Glasgow, UK; 5 Division of Signal Transduction and Therapy, University of Dundee, Dundee, UK; 6 FingerPrints Proteomics Facility, University of Dundee, Dundee, UK

**Keywords:** Phosphorylation, Lung cancer, Breast cancer, Lymphoma, Biomarker

## Abstract

**Background:**

Cyclin-dependent protein kinase-5 (CDK5) is an unusual member of the CDK family as it is not cell cycle regulated. However many of its substrates have roles in cell growth and oncogenesis, raising the possibility that CDK5 modulation could have therapeutic benefit. In order to establish whether changes in CDK5 activity are associated with oncogenesis one could quantify phosphorylation of CDK5 targets in disease tissue in comparison to appropriate controls. However the identity of physiological and pathophysiological CDK5 substrates remains the subject of debate, making the choice of CDK5 activity biomarkers difficult.

**Methods:**

Here we use *in vitro* and in cell phosphorylation assays to identify novel features of CDK5 target sequence determinants that confer enhanced CDK5 selectivity, providing means to select substrate biomarkers of CDK5 activity with more confidence. We then characterize tools for the best CDK5 substrate we identified to monitor its phosphorylation in human tissue and use these to interrogate human tumour arrays.

**Results:**

The close proximity of Arg/Lys amino acids and a proline two residues N-terminal to the phosphorylated residue both improve recognition of the substrate by CDK5. In contrast the presence of a proline two residues C-terminal to the target residue dramatically reduces phosphorylation rate. Serine-522 of Collapsin Response Mediator-2 (CRMP2) is a validated CDK5 substrate with many of these structural criteria. We generate and characterise phosphospecific antibodies to Ser522 and show that phosphorylation appears in human tumours (lung, breast, and lymphoma) in stark contrast to surrounding non-neoplastic tissue. In lung cancer the anti-phospho-Ser522 signal is positive in squamous cell carcinoma more frequently than adenocarcinoma. Finally we demonstrate that it is a specific and unusual splice variant of CRMP2 (CRMP2A) that is phosphorylated in tumour cells.

**Conclusions:**

For the first time this data associates altered CDK5 substrate phosphorylation with oncogenesis in some but not all tumour types, implicating altered CDK5 activity in aspects of pathogenesis. These data identify a novel oncogenic mechanism where CDK5 activation induces CRMP2A phosphorylation in the nuclei of tumour cells.

**Electronic supplementary material:**

The online version of this article (doi:10.1186/s12885-015-1691-1) contains supplementary material, which is available to authorized users.

## Background

CDK5 is a Serine/Threonine. protein kinase belonging to the CMGC subfamily. CDK5 is the catalytic subunit of an active heterodimeric complex consisting of CDK5 bound to either p35 or p39, two similar CDK5 cofactors encoded for by different genes (*CDK5R1* and *CDK5R2*) [1, 2]. These regulatory subunits have little primary sequence homology to cyclins but possess domains with three-dimensional structures similar to the Cdk-binding motif of cyclins [3, 4] and are highly selective in their binding of CDK5 [5]. The levels of p35 and p39 are not regulated through the cell cycle suggesting the function of CDK5 is not related to that of its cyclin binding relatives that are crucial regulators of cell cycle progression.

Mice lacking CDK5 die just before or after birth, with serious defects in neuronal layering of many brain structures [6–8]. p35 null mice have a similar inverted cortical layering observed in the CDK5 null mouse but are viable with normal cerebellum, suggesting variable redundancy in p35 and p39 protein function across the brain [9–11]. The p35 null mice exhibit increased susceptibility to seizures, while the p39 null mice have little apparent deficit, which may suggest that p35 is the more dominant regulator of CDK5 activity. Meanwhile, mice lacking both p35 and p39 have a very similar phenotype to that of the CDK5 null mouse providing evidence that p35 or p39 regulation of CDK5 is required for development of the brain [12].

As such, CDK5 has predominantly been studied in post-mitotic neurons, the major site of expression of p39 and p35. The main mode of CDK5 regulation in neurons is currently thought to be modulation of the expression or stability of p35 and p39. The proteolytic clipping of these proteins by the calcium regulated protease calpain produces p25 and p29, respectively [13, 14]. This alters the subcellular localization of the p25/p29 proteins, and the associated CDK5 catalytic subunit, since the N-terminal portion of p35/p39 that is lost contains a membrane localization domain. p25 is reported to be more stable than p35, and p25/CDK5 complexes are reported to contain intrinsically higher activity [15], which would have obvious implications on CDK5 substrate phosphorylation in diseases with altered p35-p25 ratio. However the relevance of p35 to p25 ratio on steady state CDK5 substrate phosphorylation, and subsequent disease development remains to be fully appreciated.

There is a diverse array of proposed substrates of CDK5, although most have not been validated as true physiological substrates *in vivo* or even in intact cells. Most substrates of CDK5 identified to date have key neuronal functions. These include tau [16, 17], and CRMP2 [18–20], with hyperphosphorylation of these proteins being associated with the generation of neurofibrillary tangles, one of the two hallmarks of Alzheimer’s disease. The phosphorylation of Pctaire 1, spinophilin, axin and neurabin 1 by CDK5 regulates the development of dendritic spines and axons [21–23] while NMDA receptor activity is increased through the phosphorylation of its NR2A subunit by CDK5 [24], and dopaminergic signalling is controlled by CDK5 through the phosphorylation of dopamine cAMP-regulated phosphoprotein of 32 kDa, DARPP32 [25]. This substrate profile reflects the neuronal focus of CDK5 research and, combined with the lack of cell cycle regulation of its activity, means that CDK5 has generally not been associated with a key role in cancer initiation, progression or therapy. However, more ubiquitous cell regulatory actions of CDK5 outside of the brain are well described [26, 27]. In addition there are many lines of evidence linking CDK5 to growth and cancer related actions. These include; i) the phosphorylation of oncogenic proteins such as Rb [28], ATM [29], Bcl-2 [30], p53 [31], STAT3 [32], and talin [33], ii) the observed dysregulation of CDK5 activity in leukaemia [34] and pancreatic carcinoma cells [35, 36], iii) a significant correlation between the expression of p35/CDK5 and the degree of differentiation and metastasis in non-small cell lung cancer [37], as well as increased expression and activity of CDK5 in human hepatocellular carcinoma (HCC) [38], iv) an association between polymorphisms in the CDK5 promoter and lung cancer risk in a specific Korean population [39], v) the demonstration that CDK5 activation enhances medullary thyroid carcinoma (MTC) in a conditional mouse model [40, 41], while inhibition of CDK5 activity reduces tumour growth, motility and metastasis in pancreatic cancer cells [35] [42, 43], and ablation/inhibition of CDK5 significantly decreased HCC cell proliferation [38].

All of the above data suggests abnormal activation of CDK5 increases the risk of, or aggressiveness of, specific forms of cancer. However there are also reports that pharmacological (roscovitine) or siRNA inhibition of CDK5 enhances the proliferation of the breast cancer cell lines MCF-7 and MDA-MB321, while application of carboplatin, a chemotherapeutic used in the treatment of breast cancer, induces CDK5 activation [44]. Similarly, CDK5 levels decrease in gastric cancer and its nuclear accumulation suppresses gastric tumorigenesis [45].

Although this indicates a complex relationship between CDK5 activity and growth of different cancer types, the general theme is that tight regulation of CDK5 activity is important for normal cell physiology and that localised or temporal gain (or loss) of function is associated with abnormal cell proliferation. This complex relationship makes it vital to develop the means to accurately assess CDK5 activity in tissue to clarify the potential contribution that this kinase plays in tumourigenesis and whether it presents any novel opportunities for intervention.

The aims of our study were to identify high-confidence substrates as biomarkers of CDK5 activity in tissue and use these surrogate marker(s) of CDK5 activity to establish whether CDK5 activity was altered in human carcinoma.

## Methods

### Materials

Peptides (Additional file 1: Table S1) were synthesized by Pepceuticals Ltd, Enderby, Leicestershire UK. Active forms of the CMGC protein kinases were purchased from MRC Protein Phosphorylation Reagents, University of Dundee, except for p35/CDK5 and p25/CDK5 (Millipore UK Ltd, Herts, UK).

Antibodies: The pCRMP2 Ser522 and pCRMP4 Ser522 were generated in-house as previously described [20] and are available from MRC Protein Phosphorylation Reagents, University of Dundee (mrcppureagents.dundee.ac.uk), while the pTau S202 (Cell Signalling, catalog. No.11834), pTau T205 (Invitrogen, catalog. No.44-738G), and pTau S235 (Bioworld, catalog. No.BS4193) antibodies were commercially available.

DNA Constructs: The generation of the expression constructs for human CRMP proteins have been described previously [20], while human tau expression constructs were obtained from MRC Protein Phosphorylation Reagents, University of Dundee. Expression constructs for CDK5, p35 and p25 were generated by Dr Margereta Nikolic, Imperial College, London.

### Cell culture

Embryonic primary cortical neurons were isolated from Sprague–Dawley rats at day 18 gestation. Briefly, following dissection, cortex was digested in 0.25 % trypsin in Hank’s balanced salt solution at 37 °C for 20 min. Cells were manually dissociated by trituration using a fire-polished Pasteur pipette and plated onto 0.01 % poly-l-lysine-coated coverslips at a density of 2–5 × 10^6^ cells per 6 cm well, then incubated at 37 °C with 5 % CO_2_ in Neurobasal medium (Gibco) containing 2 % (vol/vol) B27 serum replacement (Invitrogen), penicillin (Sigma; 100 units/ml), streptomycin (Sigma; 100 μg/ml), and 1 % (vol/vol) L-glutamine (Sigma). HeLa and tumour cell lines were maintained in DMEM supplemented with 4.5 g/L glucose, 10 % (vol/vol) FCS, 1 % (vol/vol) penicillin (100 units/ml)/streptomycin (100 μg/ml) at 37 °C in 5 % CO2.

Plasmids were introduced into cells using Lipofectamine 2000 (Invitrogen) as per manufacturers instructions. Cells were incubated for 4 h at 37 °C before the transfection medium was removed and replaced with 5 ml growth medium. Cells were then incubated overnight at 37 °C, prior to lysis or fixation as below.

### Cell lysis for protein isolation

Cells were lysed in ice-cold lysis buffer (1 % (v/v) Triton X-100, 50 mm Tris–HCl, pH 7.5, 0.27 M sucrose, 1 mM EDTA, 0.1 mM EGTA, 1 mM sodium orthovanadate, 50 mM sodium fluoride, 5 mM sodium pyrophosphate, 0.1 % (vol/vol) β-mercaptoethanol, and Complete protease inhibitor tablet (1 per 10 ml, Roche Applied Science, Basel, Switzerland)). Following centrifugation to remove insoluble material, supernatants were collected, and protein concentrations determined using the Bradford method.

### Immunofluorescence

Neurons were fixed in 4 % (w/v) paraformaldehyde in PBS for 10 min at 4 °C, permeabilised with 0.1 % (v/v) Triton X-100 in TBS for 3 min at room temperature, blocked with 1 % (w/v) BSA in TBS containing 0.005 % (v/v) Tween-20 for 1 h at room temperature, and incubated with primary antibodies diluted 1:50 in PBS containing 5 % (w/v) BSA for 1 h at room temperature. Secondary antibodies conjugated to Cy-3 fluorophores were diluted 1:250 in PBS containing 5 % (w/v) BSA and incubated with neurons for 1 h at room temperature. Neurons were counterstained with 0.5 ug/mL DAPI solution (Invitrogen). Image acquisition was performed on a Leica SP-5 laser scanning confocal imaging system using 63× objectives.

### Immunohistochemistry

Ethical approval was obtained by review through the Tissue Access Committee of Tayside Tissue Bank (approval # TR338) and the studies follow the Guidelines of the Declaration of Helsinki for the use of human tissues for research. Sections of formalin-fixed, paraffin embedded tissue were cut at a thickness of 4 μm, collected onto Polysine-coated microscope slides (VWR International) and dried overnight at 37 °C. Sections were dewaxed in Histoclear, rinsed in alcohol and endogenous peroxidase was quenched with 0.5 % hydrogen peroxide (100 volumes) in methanol at room temperature for 35 min. After washing in water, antigen retrieval was performed by boiling sections in 10 mM citrate buffer, pH 6.0 for 15 min in a microwave. After cooling, sections were rinsed in PBS and blocked with 5 % normal serum in PBS containing 5 % (v/v) avidin (Vector Laboratories, Peterborough, UK) for 30 min at room temperature. Sections were washed in PBS and incubated with primary antibody in 5 % normal serum in PBS containing 5 % (v/v) biotin at 4 °C overnight. After washing in PBS, sections were then incubated with biotinylated secondary antibody (1:250) (Vector Laboratories) for 30 min at room temperature, followed by streptavidin complexed with biotinylated peroxidase (Vectastain ABC kit; Vector Laboratories) at room temperature for 30 min. The peroxidase complexes were visualized using 0.25 mg/ml diaminobenzidine tetrahydrochloride (DAB) (Sigma) in PBS containing 5 mM imidazole (pH 7.0) and 0.075 % hydrogen peroxide for 10 min at room temperature. Cell nuclei were counterstained with haematoxylin (Sigma), dehydrated through graded alcohols, cleared in HistoClear and mounted in DPX. Images were taken using a Spot Insight QE digital camera or slides were digitally scanned (x40) using an Aperio ScanScope XT.

### Cell fractionation

Adherent cells (1–10 × 10^6^ cells) were harvested in 0.05 % (w/v) trypsin-EDTA and pelleted at 500× g for 5 min, washed 2x in PBS before subcellular fractionation which was preformed to the manufacturer’s specifications (Thermo Scientific- Cell fractionation Kit).

### Assay of purified protein kinase activity

Specific activity (pmol/min) was determined for all protein kinases by incubating known amounts of kinase (0.01-1 μg) with the generic substrate myelin basic protein (MBP, 0.3 mg/ml final) in kinase buffer (25 mM MOPS pH 7.5, 0.05 % (v/v) Brij-35, 0.25 mM EDTA, 5 % (v/v) glycerol) plus 10 mM MgCl2, and 100 μM [γ-32P] ATP (approx 0.5 × 10^6^ CPM/nmol) as previously described [46]. Peptide kinase assays were performed with 2mUnits of each kinase as above, except MBP was replaced with the peptide at the concentration given in figure legends. One unit of activity of each protein kinase was calculated as 1 nmole of phosphate transferred/min.

### Phosphorylation of protein substrates

Recombinant protein substrates were incubated with 2mUnits of each CMGC kinase as for MBP above for the times and at the concentrations given in figure legends. Reactions were terminated by the addition of SDS-PAGE loading buffer and heating to 70 °C for 15 mins. Aliquots were subjected to SDS-PAGE, stained with Coomassie Brilliant Blue (CBR-250), the gels were dried and radiolabeled bands visualized by autoradiography. Quantification of nmoles of phosphate incorporated was obtained by excising the stained protein band from the gel and counting in scintillation fluid.

### Western blotting

SDS loading buffer was added to cell lysates and samples subjected to electrophoresis on 4-15 % polyacrylamide gels (Invitrogen) prior to transfer to nitrocellulose using the XCell II blot module (Invitrogen). Blots were blocked in 5 % (w/v) milk in TBST (50 mM Tris HCl pH7.4, 150 mM NaCl, and 0.1 % (v/v) Tween-20) and incubated overnight at 4 °C with the primary antibody diluted in 5 % (w/v) milk in TBST. Blots were washed in TBST and bound antibodies were detected using secondary antibodies linked to a fluorescent conjugate dye. Blots were visualized using a LICOR Odyssey® Infrared Imaging System (LICOR, Lincoln, NE).

### Mass Spectroscopy

GST-tau (0.5 μM) was incubated with either p25/CDK5 or p35/CDK5 and MgATP for 5, 20 or 60 mins. Reactions were stopped by addition of 4× SDS-PAGE sample buffer prior to alkylation. GST-tau was isolated by SDS-PAGE, identified by coomassie staining and the destained protein band digested with 0.1 ml 2 g/ml trypsin in 50 mM TEAB overnight. Digests were extracted with 0.1 ml acetonitrile, supernatants dried, dissolved in 0.1 ml 5 % acetonitrile/0.25 % FA and 15 μl of sample from each time point separated on a 150 x 0.075 mm nanoC18 HPLC column prior to analysis on an Orbitrap-velos mass spectrometry system as described previously [47]. LC-MS data was searched against Uniprot database using Mascot 2.4 and interrogated using Proteome Discoverer 1.4. Quantification of the identified phosphopeptides by generating extracted ion chromatograms was performed using Xcalibur 2.2 software.

Nuclear lysates were isolated as described above, and aliquots alkylated prior to separation by SDS-PAGE and either coomassie staining or western blot (with phospho specific antibodies to CRMP2 to identify CRMP2A). The protein band equivalent to the molecular mass of CRMP2A was excised and the destained protein band digested with 20 μl 12.5 μg/ml trypsin (Roche, Sequencing Grade) in 20 mM ammonium bicarbonate overnight at 30 °C. To each digest 20 μl of 100 % acetonitrile was added and incubated for 15 min then the supernatant removed. 30 μl of 5 % formic acid was then added to each gel piece and incubated for 15 min prior to the addition of an equal volume of 100 % acetonitrile (2.5 % formic acid final concentration). This extract was then removed and pooled with the original extract from the digest. A further 10 μl of 100 % acetonitrile was added to each and incubated for 10 min prior to pooling with the previous 2 extracts. The pooled extracts were then dried down, resuspended in 10 μl of 5 % formic acid then diluted to 1 % prior to injection. 15 μl of sample from each time point was separated on a PepMap RSLC C18, 2 μM column (75 μM × 50 cm nanoViper) (Thermo Scientific) connected to an Ultimate3000 RSLCnano System (Thermo Scientific) coupled to a LTQ Orbitrap Velos Pro (Thermo Scientific) via a EasySpray source Thermo Scientific). Orbitrap Velos Pro .RAW data files analysed with Proteome Discoverer (Ver. 1.4.1) using Mascot (Ver. 2.4.1) as the search engine against the IPI Human Database and sequence of CRMP2A.

### Statistical analysis

All statistical analysis was performed using Prism 6.0 software (GraphPad software, CA, USA). Calculation of the mean was used to determine central tendency and standard error of the mean was calculated to quantify the precision of the mean. For comparison of substrate phosphorylation following transfection of p35/CDK5 and p25/CDK5 with untransfected control, statistical analysis was performed by one-way analysis of variance (ANOVA) with Tukey’s post hoc test as comparisons between each group. For comparisons between squamous cell carcinoma and adenocarcinoma, a student’s *t*-test was performed. A p value of <0.05 was considered significant and p values are expressed in relevant figures using asterisks where * represents <0.05, ** represents <0.001, and *** represents <0.0001.

## Results and discussion

### Oncomine

As an initial assessment of the potential roles of CDK5 in human cancers, we interrogated Oncomine (https://www.oncomine.org/) and COSMIC (https://www.sanger.ac.uk/research/projects/cancergenome/) to search for evidence of differential expression or mutation of *CDK5* and its two partners *CDK5R1* (p35) and *CDK5R2* (p39) in a range of cancer types. In the Oncomine database of gene expression profiles, *CDK5* mRNA levels are lower in brain cancers compared to normal brain tissues and higher in a variety of other cancer types, notably breast, lung and lymphoma compared to the corresponding normal tissues. *CDK5R1* shows a similar pattern in these cancers, whereas *CDK5R2* is not commonly altered (Table 1). In the COSMIC database, mutations or copy number changes in any of these genes occurs very rarely (33/15583 for *CDK5*; 42/15259 for *CDK5R1* and 30/15259 mutations for *CDK5R2*; all <0.3 %; accessed 3^rd^ March, 2015). This suggests that genetic alteration and/or gene expression changes in CDK5 or its cofactors are not a common cause of, or contributor to, oncogenesis. However this does not rule out disease-related post-translational alteration in CDK5 activity, and thus we decided to investigate CDK5 substrate measurements as a means to assess CDK5 activity in human tumours.

**Table 1:**
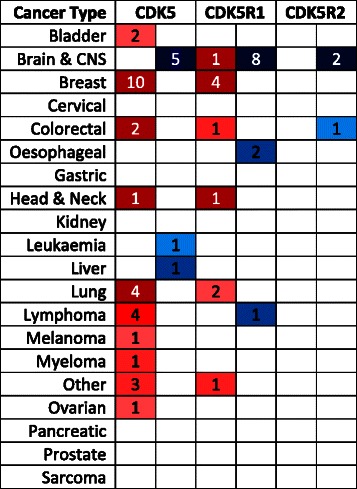
Summary of expression changes of *CDK5*, *CDK5R1* and *CDK5R2* in human cancers

Red boxes indicate the number of studies showing increased mRNA levels and blue reduced mRNA levels. Shading reflects the extent of altered expression compared to the corresponding normal tissues

### CDK5 substrates as biomarkers

**Fig. 1:**
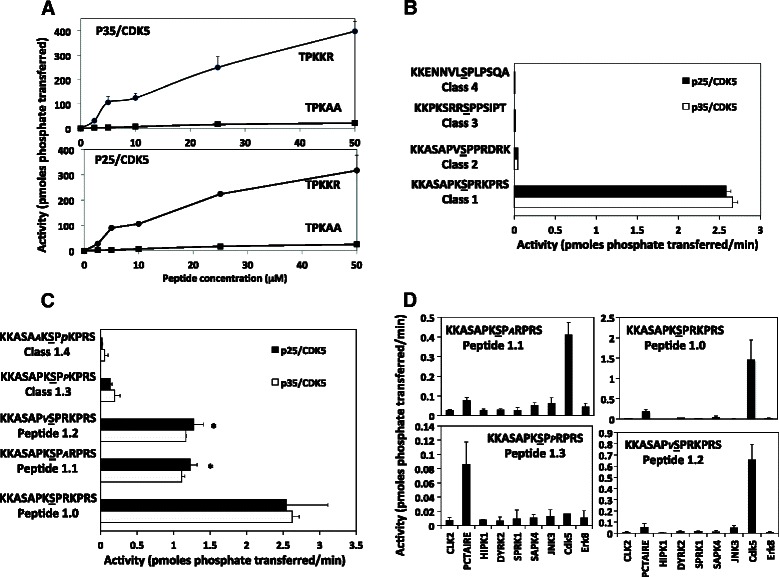
*In vitro* analysis of primary sequence determinants for p35/CDK5 and p25/CDK5. **a** The contribution of C-terminal Arg/Lys residues to recognition and phosphorylation by each CDK5 complex was assessed by incubating the peptides at the indicated concentrations with 2 m units of each CDK5 complex for 20 mins and measuring phosphate transferred to each peptide. **b** Each CDK5 complex was incubated for 20 mins with 100 μM of the indicated peptides representing the primary sequence of the 4 classes of CDK5 substrates. The phosphorylated residue is underlined. **c** To assess the contribution of specific residues ±2 positions from the target residue a further 4 peptides (at 100 μM) were incubated with 2mUnits of each CDK5 complex for 30 mins. The residue changed from the parent sequence (peptide 1.0) is in italics in each case. *indicates *p* < 0.05 compared to peptide 1.0. **d** The rate of phosphorylation of the indicated peptides by several members of the CMGC family of kinases was compared by incubating each peptide (at 50 μM) with 2 m units (as determined against the generic substrate MBP) of each kinase for 30 mins and phosphate transfer measured. In all figures the data is presented as the average of at least two different experiments performed in duplicate ± the SEM, and is given as total picomoles transferred during the assay (**a**) or pmoles transferred/min (**b**-**d**)

Previous work demonstrated that CDK5 has an absolute requirement for a Proline (Pro) residue immediately C-terminal (+1) to the phosphorylation site of its substrates, while the presence of a C-terminal Arg/Lys residue (+3 > +4 > +2) enhances phosphorylation of substrates even further [48]. Meanwhile investigation of the sequence of CDK5 substrates proposed in the literature indicates that although all contain the Pro at +1 the presence/position of a C-terminal Lys/Arg residue is quite variable (Table 2). This questions the confidence in Arg/Lys residues to the prediction of substrate recognition for CDK5, or the accuracy of some of the proposed substrates in the literature. Therefore using peptide assays we re-investigated the importance of Arg/Lys residues around the substrate phosphorylation site for both p35/CDK5 and p25/CDK5. Firstly we confirm that the presence of a Arg/Lys residue at +3 and/or +4 is a major enhancer of substrate recognition for both CDK5 complexes, at least *in vitro* (Fig. 1a). CDK5 isoforms phosphorylated a peptide containing the sequence TPKRR at a much higher rate than one with the sequence TPKAA (where the phosphorylated residue is underlined, Fig. 1a). Indeed the latter peptide is an extremely poor substrate for both p35/CDK5 and p25/CDK5 (Vmax/Km <0.05, compared to 0.6-0.8 for TPKKR), suggesting for the first time that the presence of a single Arg/Lys residue at +2 is relatively poor at conferring CDK5 recognition (Fig. 1a). This questions the validity of a number of the proposed CDK5 substrates in the literature (Table 2). Indeed one can find examples of 4 different classes of CDK5 substrate, 1) Arg/Lys residues both N- and C-terminal to the phosphorylated residue (±5), 2) Arg/Lys residues solely C-terminal, 3) Arg/Lys residues solely N-terminal and 4) no Arg/Lys residues either side of target residue. We synthesised 4 peptides representing each class of substrate sequence that resemble proposed CDK5 substrates, and incubated them with each CDK5 complex (Fig. 1b). In this case Class 1 peptides (with Arg/Lys residues both N- and C- terminal to the phosphorylated residue) were much better substrates than peptides based on other substrate classes (Fig. 1b). Surprisingly the class 2 peptide, with two C-terminal Arg/Lys residues (at +3 and +5), but none N-terminal, was a relatively poor substrate in comparison to the class 1 peptide (that contained three C-terminal (+2, +3 and +5) and also one Arg/Lys residue N-terminal (at −1)). Peptides lacking a C-terminal Arg/Lys residue (class 3 and 4) were not phosphorylated by either CDK5 complex, even when N-terminal Arg/Lys residues were present (Fig. 1b). This data suggests that multiple C-terminal Arg/Lys residues greatly enhance phosphorylation by CDK5 with those at +3/4 being crucial for recognition by CDK5.

**Table 2:** Comparison of primary structures of proposed CDK5 substrates

Substrate	Phosphorylated sequence	Proposed effect of phosphorylation
CLASS 1 (Lys/Arg residues N- and C-terminal)		
CRMP2	PASSAKT*S*PAKQQ (S522)	Axon Growth and development
CRMP4	PAGSARG*S*PTRP (S522)	Myelin dependent axon outgrowth
Synaptojanin	EAPK*S*PGTTRKD (S1144)	Inhibits interaction with endophilin 1
Tau	TPPK*S*PSSAKS (S235/AT180)	Makes tau a better substrate for GSK3
Tyr Hydroxylase-1	YTPTPR*S*PRFIGRR (S31)	Makes protein more stable
ATM	CLCIHTKH*T*PNKIAS (mouse S793)	Activation of ATM- apoptosis
Bcl-2	EMAART*S*PLRPLV (mouse S80)	Induces neuronal survival
Cdh-1	SQKLLR*S*PRKPTRK (mouse S163)	Stabilises cyclin B1
mSDS3 (HDAC)	NKLK*S*PKRPASPSS (mouse S224)	Promotes dimerisation and activation
PIK-A	RGKL*S*PRKGKSKTL (mouse S279)	Stimulates PI 3-kinase/Akt pathway
Neurabin I	GKGGHS*S*PQRRMKPKEF (S95)	Regulates binding to F-actin
CLASS 2 (Lys/Arg residues C-terminal only)		
DARPP32/PPP1R1B	PCAY*T*PPSLK (T75)	Phospho-DARPP32 inhibits PKA
Inhibitor-1	MEQDN*S*PRKIQFTVP (S6)	Regulates activity toward PP1
Dynamin I	SPTSSP*T*PQRRAPA (T778)	Regulates endocytosis
Inhibitor-1	TLAM*S*PRQRKKMTRITP (S66)	Regulates activity toward PP1
SPAR	LGAATS*S*PRSGPGKE (S1328)	Regulates synaptic plasticity
Cdh-1	ANSPVS*S*PSKHGDR (mouse S40)	Stabilises cyclin B1
Mef2A	SEPI*S*PPRDRMTP (mouse 406)	Regulates apoptosis
Mek1	GDAAE*T*PPRPRTP (mouse T283)	Inhibits MEK signalling
p53	CTSA*S*PPQKKKPL (mouse S314)	Regulates apoptosis of PC12 cells
PLD2	FAVTH*S*PAREAA (mouse S134)	Activation (part of EGF action)
STAT3	IDLPM*S*PRTLDS (mouse S727)	Upregulated transcriptional activity
Stathmin (Leukemia-associated phosphoprotein p18)	VPDFPL*S*PPKKKD (mouse S41)	Stabilises protein
Talin	EDSV*S*PKKSTVLQ (mouse S425)	Regulates binding to smurf1/cell migration
CLASS 3 (Lys/Arg residues N-terminal only)		
Doublecortin	STPKSKQ*S*PISTPT (mouse S332)	Unclear
CLASS 4 (No Lys/Arg residues)		
FAK	EGFYP*S*PQHMVQT (mouse S766)	Critical for neuronal migration
p53	PEDILP*S*PHCMDDL (mouse S33)	Regulates apoptosis of PC12 cells
PSD95	LPNQAN*S*PPVIV (S35)	Regulates structure of synapse

Primary sequence surrounding proposed CDK5 target residues; substrates are classified by presence of Arg/Lys residues (underlined) within 5 amino acids N-terminal or C-terminal to the *phosphorylated residue* which is always N-terminal to a proline

We extended this comparison to peptides where the number and position of the Arg/Lys residues varied (Fig. 1c). The class 1 peptide (Lys/Arg at −1, +2, +3, and +5, peptide 1.0) was phosphorylated at twice the rate to one where the +2 Arg/Lys residue was replaced by Ala (peptide 1.1), or the −1 Arg/Lys residue was replace by Val (peptide 1.2). Peptide 1.3 was identical to peptides 1.0 and 1.1 except the +2 residue was a Pro (a modification which may have rendered peptide 2 in Fig. 1b a poor substrate). This single amino acid change reduces the rate of phosphorylation by >90 % compared to peptide 1.0 and 1.1 (Fig. 1c). Interestingly the substrates Mef2A, MEK1, stathmin, DARP32, PSD95 and p53 (at Ser314) have a Pro at this position relative to their proposed phosphorylation site, implying that these proteins should be relatively poor CDK5 substrates (Table 2). Indeed we confirmed that Mef2A and p53 are very poor substrates for CDK5, *in vitro* at least (Table 3). Interestingly none of the class 1 substrates listed in Table 2 have a Pro at position +2.

**Table 3:** Comparison of phosphorylation rates of proposed CDK5 substrates

	*p35/Cdk5*			*p25/Cdk5*		
	Vmax	Km	Vmax/Km	Vmax	Km	Vmax/Km
CRMP4	0.665	0.401	1.660	0.155	0.252	0.6151
CRMP1	0.735	1.260	0.594	0.191	1.458	0.131
CRMP2	0.722	1.650	0.438	0.179	1.660	0.108
Tau	0.630	1.890	0.3347	0.261	1.419	0.186
Dynamin 1	0.309	2.780	0.111	0.187	2.040	0.092
p53	ND	ND	ND	ND	ND	ND
PPARγ	ND	ND	ND	ND	ND	ND
Mef2a	ND	ND	ND	ND	ND	ND

Phosphorylation kinetic parameters were established during initial rate conditions by incubating equal amounts of p35/Cdk5 and p25/Cdk5 with varying concentrations of the indicated substrates and Mg [γ-32P] ATP for 10 min at 30 °C. Vmax and Km values were calculated using the Lineweaver-Burk equation. Vmax values are in pmol/min and Km are in μM. Values are representative of at least two independent experiments performed in duplicate. ND; not determinable, these substrates were not phosphorylated to a significant level under these conditions

This dramatic negative effect of a Pro substitution at +2 prompted us to investigate whether the presence of three Pro residues in close proximity to the phosphorylation site in peptide 1.3 worsened the rate of phosphorylation rather than the specific position. We replaced the Pro at −2 in peptide 1.3 with Val to generate peptide 1.4 in Fig. 1c. However this modification did not restore phosphorylation of the peptide by CDK5 (peptide 1.0-Pro vs 1.4-Lys), indicating the Pro at +2 is a novel inhibitory structural feature in substrate recognition for CDK5.

Finally we incubated these peptides with several other members of the CMGC protein kinase family in order to investigate the selectivity of the Arg/Lys and proline residue features identified for CDK5 (Fig. 1d). Peptide 1.0 is highly selective for CDK5, with only PICTAIRE showing any ability to phosphorylate this peptide to any significant level (<80 % that of a matched amount of MBP kinase activity of CDK5). Similarly, CDK5 is the most effective kinase at phosphorylating peptides 1.1 and 1.2 (Fig. 1d). However, PICTAIRE phosphorylates peptide 1.3 (with Pro at +2) to a greater extent than CDK5 (or any other CMGC kinase tested, Fig. 1d), suggesting that substrates with a S/TPP sequence are likely to be better PICTAIRE targets than CDK5 (such as Mef2A and p53).

In summary this data identifies four novel aspects of CDK5 substrate recognition, firstly that N-terminal Arg/Lys residues can enhance phosphorylation by CDK5, secondly that multiple C-terminal Arg/Lys residues improve the phosphorylation rate, thirdly that a Pro at +2 antagonises phosphorylation by CDK5 and finally that a Pro at −2 enhances recognition by CDK5 compared to an Arg/Lys residue. There were no differences in the rates of peptide phosphorylation between each CDK5 complex *in vitro* and we propose that peptide 1.0 is a relatively selective substrate to distinguish between CDK5 and other members of the CMGC kinase family.

These primary sequence determinants focused our attention on class 1 substrates for assessing CDK5 activity in cells and tissues.

### CDK5 substrate phosphorylation in vitro

**Fig. 2:**
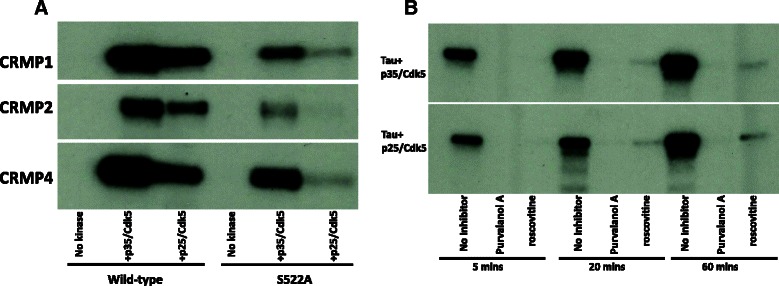
Analysis of CRMP and tau phosphorylation by CDK5 in vitro. Recombinant protein substrates (0.5 μM final concentration) were incubated with 2mUnits of each CDK5 complex and MgCl2 and [γ-32P] ATP (approx 0.5x10^6^ CPM/nmole) for 30 mins. Reactions were terminated by the addition of SDS-PAGE loading buffer and heating to 70 °C. Aliquots were subjected to SDS-PAGE, stained with Coomassie Brilliant Blue (CBR-250), the gels were dried and radiolabeled bands visualized by autoradiography. **a** Comparison of CRMP-1, −2 and −4 (wild-type and Ser522Ala mutants) phosphorylation by each CDK5 complex. **b** GST-tau (0.5 μM final concentration) was incubated with 2mUnits of each CDK5 complex for 30 min, with or without a 30 min pre-incubation with the Cdk inhibitors roscovitine (10 μM) or purvalanol A (10 μM). Data is representative of at least three different experiments

We compared the rate of phosphorylation of collapsin response mediator proteins (CRMP) by each CDK5 complex (Fig. 2a). Three members of the CRMP family are class 1 CDK5 substrates [18]. Both CDK5 complexes phosphorylate CRMP1, CRMP2 and CRMP4, and this is reduced by >75 % in the Ser522Ala mutant of each CRMP (Fig. 2a), indicating that Ser522 is the major site for phosphorylation by CDK5 in this substrate. In addition, each CDK5 complex phosphorylates tau protein *in vitro* (Fig. 2b), and this activity is completely blocked by the inclusion of either of two CDK inhibitors. Mass Fingerprinting found the major purvalanol A- and roscovitine-sensitive phosphorylation site on tau is Ser-235 (a class 1 site), with minor phosphorylation of Ser202/Thr205 (Additional file 2: Figure S1a). This was subsequently confirmed by immunoblot using site-specific antibodies (Additional file 2: Figure S1b). The high preference for Ser235 was unexpected as previous studies had indicated that CDK5 phosphorylates numerous residues on tau [49]. For example, Ser231 was reported as a CDK5 target and was not found in our studies, yet Ser231 was phosphorylated by GSK3 after tau phosphorylation at Ser235 by CDK5 (Additional file 2: Figure S1b). Therefore phosphorylation at Ser235 turns tau into a substrate for GSK3 at Ser231 making this site look like a CDK5 site in cells. Importantly >80 % of the phosphate incorporation (at least after 60 min incubation with CDK5 *in vitro*) is accounted for by Ser235/Ser202/Thr205 phosphorylation. This does not rule out additional sites on tau are phosphorylated in longer incubations, or *in vivo*. However we focused subsequent studies on tau phosphorylation by CDK5 only at these three residues. We investigated several additional proposed CDK5 substrates (Table 3), however in comparison to CRMPs and tau these were relatively poorly phosphorylated *in vitro*, making confirmation of phosphorylation site difficult. Hence we focused on establishing whether these specific sites identified on CRMP or Tau could be developed as markers of CDK5 activity in cells or tissues.

### CDK5 substrate phosphorylation in cells

Human CRMP2 or CRMP4 was co-expressed with or without the CDK5 catalytic subunit and either p25 or p35 in HeLa cells (Additional file 2: Figure S2). Cells were lysed and protein lysates probed for the expression and phosphorylation of CRMP2 (Additional file 2: Figure S2a and b) or CRMP4 (Additional file 2: Figure S2c and d). Overexpression of either CDK5 complex enhances CRMP2 phosphorylation at Ser522, but in contrast Ser522 phosphorylation of CRMP4 is not influenced by overexpression of either CDK5 complex. This implies CRMP4 is not a CDK5 substrate when expressed in cells or that the Ser522 site on ectopic CRMP4 becomes rapidly and fully phosphorylated by endogenous CDK5. The induction of Ser522 phosphorylation of CRMP2 by p25/CDK5 was greater than that for p35/CDK5 however the expression of p25 was consistently greater than p35 (Additional file 2: Figure S2a and c).

When either CDK5 complex is co-expressed with tau in HeLa cells (Additional file 2: Figure S3) there is a significant increase in phosphorylation of all of the 3 sites that we identified in the *in vitro* analysis above. Interestingly these sites are poorly phosphorylated in the absence of CDK5 co-expression while the effect of p25/CDK5 was similar to that of p35/CDK5 implying no major difference in targeting by alteration of the p35-p25 ratio in cells.

*These data support the hypothesis that an increase in CDK5 expression and/or activity associated with disease would alter the phosphorylation of CRMP2 (at Ser522) and tau (at Ser235 and Ser202/Thr205), and thus these substrates are potential biomarkers of aberrant CDK5 activity.*

**Fig. 3:**
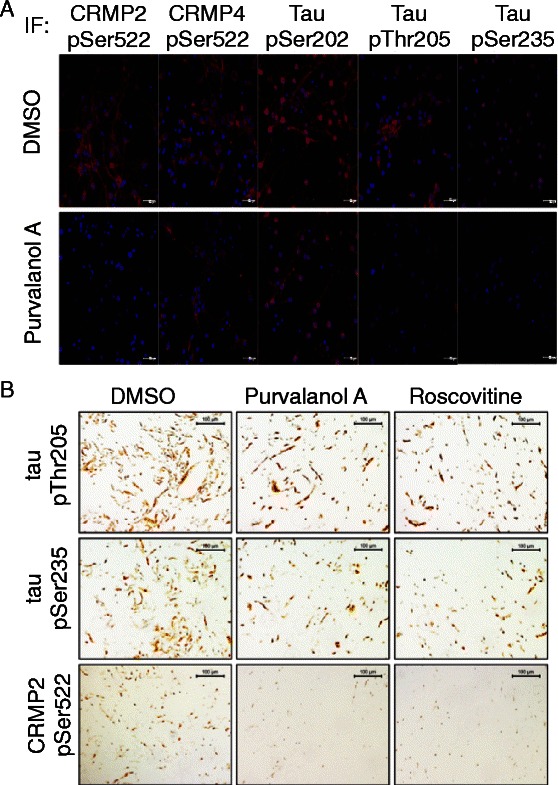
Imaging of CDK5 substrate phosphorylaiton in primary cells. Rat primary cortical neurons were cultured for 6 days *in vitro.* **a** Cells were incubated with 10 μM purvalanol A or vehicle for 3 h prior to fixation, permeabolisation and staining with the indicated antibodies. Phosphospecific antibodies were detected by Cy-3 bound 2ry antibodies (*red*) and nuclei were counter-stained with the DNA-binding dye DAPI (*blue*). *Scale bar* = 60 μm. **b** The primary neuronal cultures were incubated with either 10 μM purvalanol A, roscovitine or vehicle for 3 h prior to fixation in formaldehyde and embedding in paraffin. Sections were taken from each paraffin block and incubated with the phospho-antibodies as indicated, then biotinylated secondary antibody followed by streptavidin complexed with biotinylated peroxidases which were visualized using DAB staining. Cell nuclei were counterstained with hematoxylin and mounted in DPX. *Scale bar* = 100 μm. Images are representative of sets from at least three different neuron preparations

Next we used immunofluorescence to investigate whether our phosphospecific antibodies could selectively detect phosphorylation of endogenous CRMP or tau in primary cells (Fig. 3a). We incubated primary neurons with or without purvalanol A (CDK inhibitor) and then fixed and stained the isolated cells for substrate phosphorylation. CDK inhibition reduces CRMP2 phosphorylation at Ser522 but does not alter CRMP4 phosphorylation. Taken together with the CRMP and CDK5 co-expression data (Additional file 2: Figure S3) this indicates that Ser522 of CRMP2, but not CRMP4, is a physiological target for CDK5. This is in agreement with previous work in CDK5 null tissue where CRMP2 phosphorylation is absent yet CRMP4 phosphorylation at Ser522 persists [20].

Tau phosphorylation at Thr205 but not Ser202 is reduced by CDK inhibition (Fig. 3a). However the signal to noise ratio for the phospho-Ser235 antibody is very weak which makes assessing changes in phosphorylation of this site difficult even in cells with high levels of tau. This questions whether tau phosphorylation is a useful biomarker of CDK5 activity, however we cannot rule out that Ser235 phosphorylation could be increased in a disease specific manner, only becoming significant when CDK5 activity increased above levels detected in healthy tissue.

Next we investigated immunohistochemical staining of phospho-CRMP2 and phospho-tau after the primary neurons were embedded in paraffin to accurately mimic the fixation and processing of human clinical tissues used in diagnostic histopathology (Fig. 3b). The intensity of staining and the number of cells stained with the antibody to phospho-Ser522 of CRMP2 is reduced by CDK inhibition (by both purvalanol A and roscovitine). In contrast tau phosphorylation at Ser235 or Thr205 is not significantly affected by the inhibitor treatment (Fig. 3b).

Therefore our *in vitro* and cell based studies suggest that of all of the potential substrates examined CRMP2 phosphorylation at Ser522 is most worthy for investigation as a surrogate marker for altered CDK5 activity in tumour tissue.

### CRMP2 phosphorylation in tumours

**Fig. 4:**
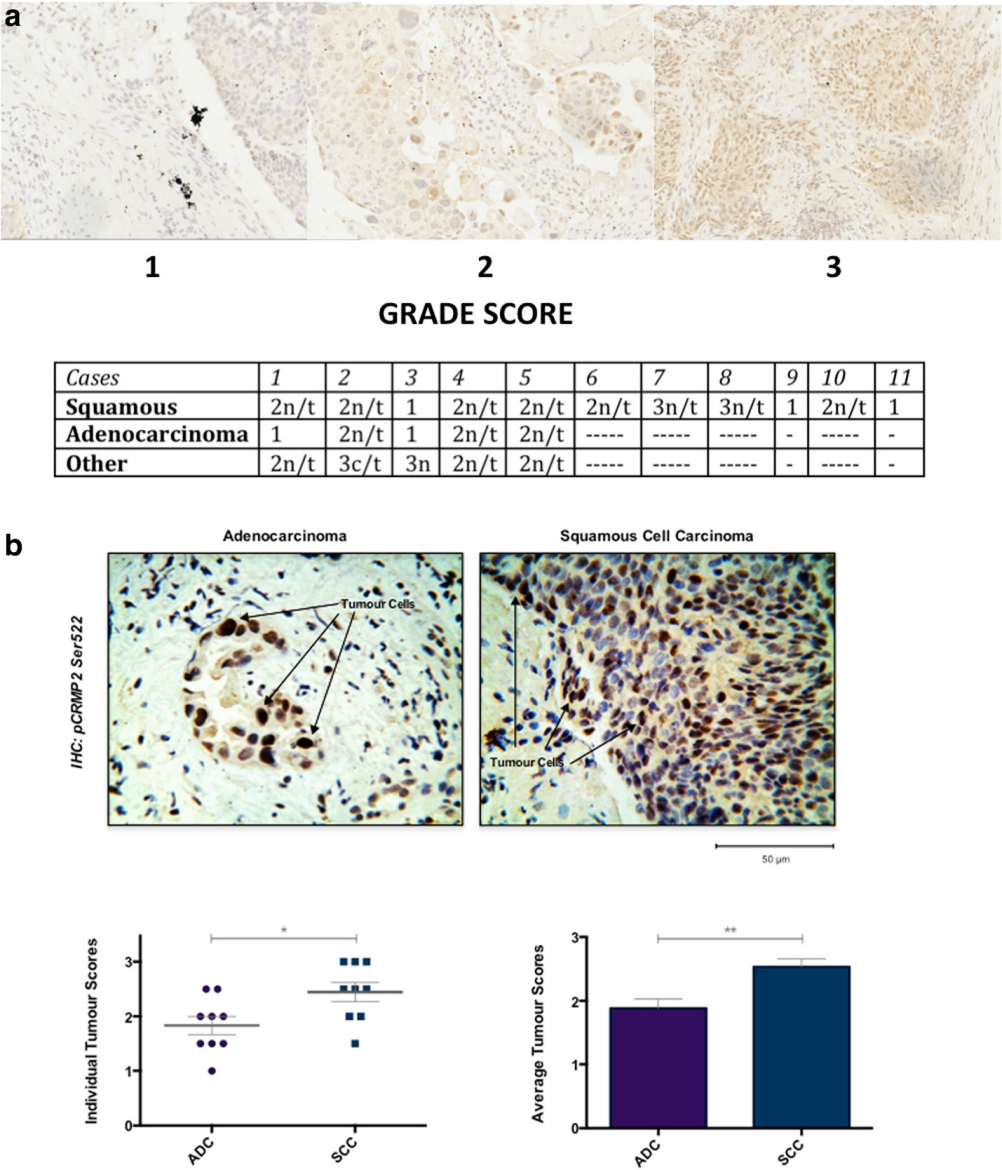
CDK5 substrate phosphorylation in human tissue. **a** Nineteen lung tumour biopsies were immunostained with the pCRMP2 S522 antibody and developed with DAB. Tumour sections were designated squamous (11), adenocarcinoma (4) or other (4) and graded using a semi-quantitative scale from 0–3, with 0 representing no staining, 1 representing light brown staining, 2 representing moderate brown staining and 3 representing dark brown staining. Representative images for each grade are provided. Clear nuclear (n) or cytoplasmic (c) staining is indicated, and slides where the staining was specific to tumour tissue rather than healthy tissue are indicted by (t). **b** A second independent cohort of adenocarcinoma (ADC) and squamous cell carcinoma (SCC) sections (9 of each type) were immunostained with the pCRMP2 Ser522 antibody and scored as in A. Representative histology for each is provided in upper panel (*Scale bar* = 50 μm), while the individual and average scores for pCRMP2 Ser522 staining is given below as mean ± S.E.M. *t*-test, *P < 0.05, **P < 0.01, ***P < 0.001

Previous work had suggested that altered expression/phosphorylation of specific CRMP isoforms was associated with lung and breast cancer [50–54]. Therefore we initially investigated biopsies from 21 different non-small cell lung cancer (NSCLC) patients for pSer522 CRMP2 staining (Fig. 4). The staining was graded by two independent pathologists using a semi-quantitative scale from 1–3, with 1 representing low intensity staining, 2 moderate staining and 3 high level staining. Staining for pSer522 is strong in the nucleus of tumour cells but is absent from the surrounding non-neoplastic epithelium (Fig. 4a and b). Interestingly this initial investigation suggested that immunopositive staining is more a feature of squamous cell carcinoma (8/11 with a score of 2 or 3, average score = 1.91) than adenocarcinoma (5 samples, none with score 3, average score = 1.8). To investigate this in more detail an additional 18 lung tumour samples were obtained containing roughly equal numbers of both tumour types in order to compare histologically graded staining between adenocarcinoma and squamous cell carcinoma. In addition, a squamous carcinoma from skin was included as this is histologically different from squamous carcinoma sourced from other areas of the body. The staining was graded as described above. Consistent with the preliminary observation, Ser522 phosphorylated CRMP2 is predominantly localised in the nuclei of cancer cells and absent from healthy tissue, confirming the association of this phosphorylation with tumourigenesis and the unusual localisation of the CRMP2 (Fig. 4c). CRMP2 phosphorylation at Ser522 has a higher intensity in squamous cell carcinoma compared to adenocarcinoma immunostaining. No phospho-Ser522 staining is observed in skin squamous carcinoma (data not shown), suggesting both selectivity in tumour reactivity, and a potential clinical utility for the pSer522 antibody to identify lung metastasis.

The oncomine data on gene expression levels indicated that *CDK5* itself is not differentially expressed in lung cancer subtypes (Table 1), but *CDK5R1* mRNA levels are higher in lung squamous cancers than adenocarcinomas. The lack of common *CDK5* mutations associated with oncogenesis is perhaps not that surprising as there are no known point mutations that promote constitutive activation of CDK5. It is not even clear if enhanced CDK5 expression would induce greater cellular CDK5 activity without a simultaneous upregulation of p35 or p39. Upregulation of CDK5 activity could occur due to changes in the expression/regulation of one or other of its regulatory subunits, but again it is not clear whether this would enhance cellular CDK5 activity without simultaneous increase in the catalytic subunit of CDK5. In addition, there are no reported point mutants in *CDK5R1 or CDK5R2* that result in significant increases in CDK5 activity. Rather the ratio of p35 to its cleavage product p25 is proposed to alter subcellular location and potentially substrate selection. This makes monitoring associations between altered CDK5 activity and oncogenesis difficult without robust markers of intracellular CDK5 activity. Interestingly, the phosphorylation of the CDK5 substrate CRMP2 is altered in lung [52, 54] and breast [53] carcinoma, however those studies implied that it was changes in Thr509/Thr514 phosphorylation of CRMP2 that associated with carcinoma. These are not CDK5 phosphorylation sites. However we have previously established that CRMP2 phosphorylation at Ser522 by CDK5 is a prerequisite for the subsequent sequential phosphorylation of CRMP2 at Ser518, Thr514 and finally Thr509 by GSK3 [20]. Thus enhanced Ser522 phosphorylation is likely to be required for induction of, and may be sufficient to induce, the phosphorylation of CRMP2 at Thr509/514. Therefore our study links, for the first time, CRMP2 phosphorylation at Ser522, the CDK5 target site, to carcinoma. This is of particular mechanistic importance since the reported increase in Thr509/514 phosphorylation implicates increased GSK3 as the potential driver leading to enhanced phosphorylation of CRMP2 in carcinoma, however our data identifying enhanced Ser522 phosphorylation indicates that this is not necessarily true. Rather, increased CDK5 would also indirectly lead to enhanced phosphorylation of Th509/514 of CRMP2 subsequent to the increase in Ser522 phosphorylation. It is quite possible that there is a synergistic effect of a combined increase in GSK3 and CDK5 activity. This has major implications for understanding the underlying biochemistry of tumours with altered CRMP2 phosphorylation and the potential development of interventions.

As changes in Thr509/514 phosphorylation of CRMP2 had been reported in more than one carcinoma we investigated the tumour selectivity of Ser522 immunostaining further in tissue microarrays (TMA), covering an additional eight human tumour types (Tables 4). There is only a single positively stained core in the control TMAs, so control tissue is deemed negative for pCRMP2 Ser522 staining. Likewise, ovarian, renal, prostate and colorectal cancers are almost completely negative for pCRMP2 Ser522 staining, with less than 1.7 % expressing immunopositive staining. Positive staining is observed in the breast TMA however this constitutes only 8 % (4/48) of patient samples and these all have a relatively low quick score of 3. Previously Shimada and colleagues reported increased phosphorylation of Thr509 of CRMP2 in the nuclei of breast carcinoma and that this increased in proportion to the histological grade and triple-negative subtype [53]. The relatively low numbers of breast samples on our TMA prevents a similar investigation in our study. A similar number of sample cores taken from follicular lymphomas are positive for pCRMP2 Ser522 staining (10 %).

**Table 4:** Quick Score = Category A + Category B (possible 0-9), number of cases with each score given in columns for each cancer type

	*Quick Score (A + B)*									
	1	2	3	4	5	6	7	8	9	Cases
*Controls*	0	1	0	0	0	0	0	0	0	14
*Ovarian*	0	0	0	1	0	0	0	0	0	79
*Kidney*	0	0	1	2	0	0	0	0	0	181
*Prostate*	0	1	1	0	0	0	0	0	0	25
*Breast*	0	0	4	0	0	0	0	0	0	48
*Colorectal Adenoma*	0	0	0	2	0	0	0	0	0	127
*Follicular Lymphoma*	0	2	1	1	0	0	0	0	0	46
*Diffuse Large B-Cell Lymphoma (DLBCL)*	0	9	10	13	5	0	0	0	0	77

Sections were stained using the anti-pCRMP2 Ser522 and visualised using DAB chromogen. The proportion of malignant cells staining positively throughout the section (termed Category A) was assigned scores from 1–6 (1 = >0 - 5 %; 2 = 6 - 20 %; 3 = 21 - 40 %; 4 = 41 - 60 %; 5 = 61 - 80 %; 6 = 81 -100 %); the average intensity of staining in malignant cells (termed Category B) was scored as 0, 1, 2, or 3, corresponding to the presence of negative, weak, moderate, and strong brown staining, respectively. Category A and Category B values were added together to produce a quick score (range from 0, negative, to 9, strong and complete). The table provides the number of cases of each tumour type with each quick score. A tumour was considered negative when all sample cores were negative, whereas > 2 positive cores was considered a positive result

Interestingly, a much higher proportion of immunopositive cells are observed in sample cores from diffuse large B-cell lymphoma (DLBCL) with almost half (48.1 %) of patient cases scoring positive and half of those with a quick score >3 (Tables 4). This suggests that pCRMP2 Ser522 is present in more than just lung carcinoma, and is particularly abundant in DLBCL. CRMP2 has been proposed to contribute to T-lymphocyte polarisation and migration [55], and increased expression of CRMP2 in peripheral T lymphocytes is associated with their recruitment to the brain following virus-induced neuroinflammation [56]. However this is the first indication that changes in CRMP2 phosphorylation, and by implication CDK5 regulation of CRMP2, are associated with B-lymphocyte biology, in health or disease.

### Nuclear staining of CRMP2 is unusual

The immunostaining of the nucleus of tumour cells with the anti-pCRMP2 Ser522 antibody is an unexpected result as there is very limited evidence that CRMP2 enters the nucleus of cells (most work has been done in neurons). As far as we are aware there is currently only one report proposing that phosphorylated CRMP2 is in the nucleus, with phospho-509/514 of CRMP2 being detected by immunofluorescence in the nucleus of breast cancer cells [53].

**Fig. 5:**
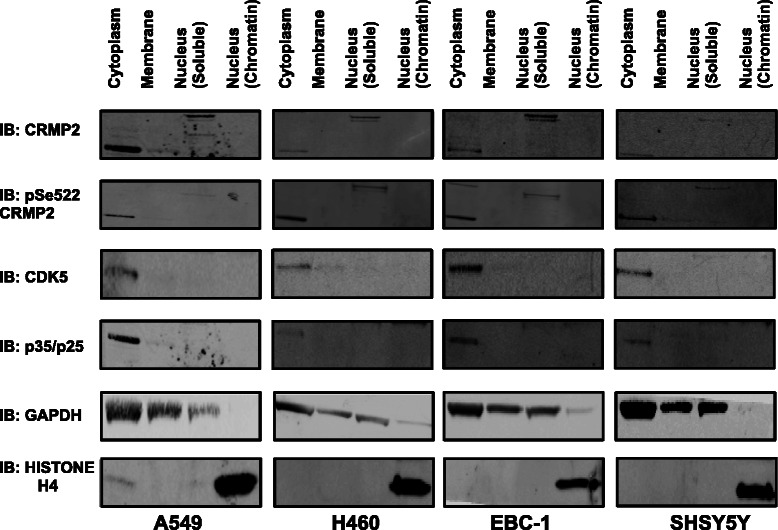
Sub-cellular localization of phosphorylated CRMP2. Subcellular fractionation of three cancer cell lines (A549, EBC-1 and H460), and a human neuroblastoma SHSY5Y cell line (positive control for CRMP2 expression) was performed prior to Western blot analysis with the indicated antibodies. GAPDH and histone H4 were used as markers for the successful fractionation of cytoplasm and chromatin-bound nuclear fraction, respectively. Western blots shown are representative of three independent experiments

To confirm the nuclear CRMP2 localisation using biochemical techniques we selected three human lung cancer cell lines representing different subtypes of NSCLC. The EBC-1 cell line is derived from human lung squamous cell carcinoma [57], the A549 cell line was initiated through explant culture of lung carcinomatous tissue and is used as a cell-based model of adenocarcinoma [58], and finally the NCI-H460 cell line which is often used as a model of large cell carcinoma [59]. We performed subcellular fractionation of these lines along with the human neuroblastoma cell line, SHSY5Y, as a positive control for CRMP2 expression (Fig. 5). The resultant nuclear, membrane and cytoplasmic protein fractions were immunoblotted using total CRMP2 and pCRMP2 Ser522 antibodies. Fractionation efficiency was assessed by immunoblot using an antibody to anti-GAPDH (cytoplasm) and anti-histone H4 (nuclear (chromatin-bound)).

The most abundant form of CRMP2 protein (62 kDa) is found only in the cytoplasmic fraction for all cell lines (Fig. 5). However, a form of CRMP2 with greater mass is detected in the soluble nuclear fraction, and this corresponded to the molecular mass of a less abundant form of CRMP2, termed CRMP2A (75 kDa) that has an N-terminal extension due to alternative splicing [60]. The CRMP2A isoform is thought to have a divergent function to the more common CRMP2 isoform, and was previously reported to be isolated to axons in neurons [60]. We detect the 75 kDa form using both the anti-pCRMP2 Ser522 and total CRMP2 antibody giving greater confidence that this is truly the CRMP2A isoform and implying that phospho-CRMP2 does exist in the nucleus of cancer cells (it was also detected in the human neuroblastoma SHSY5Y). To obtain additional evidence supporting nuclear CRMP2A localisation, peptide identification by Mass Spectrometry (1D nLC-MS/MS) of the fractionated nuclear protein lysate was performed, as this technique is independent of antibody specificity. We positively identify four peptides that correspond to human CRMP2 sequence, three of which are common to both CRMP2A and CRMP2B (IAVGSDADLVIWDPDSVK, DIGAIAQVHAENGDIIAEEQQR, NLHQGFSLSGAQIDDNIPR), but one that is only found in the N-terminal extension region of CRMP2A (IVNDDQSFYADIYMEDGLIK). This provides compelling evidence that CRMP2A is indeed present within the nucleus. Thus alternative splicing of CRMP2 regulates its nuclear localisation and it is specifically CRMP2A phosphorylation that is associated with lung, breast and lymphocyte tumour staining. This may provide the basis for development of a novel and highly selective intervention.

## Conclusions

We demonstrate that an antibody that selectively detects a validated CDK5 phosphorylation site on the substrate CRMP2 robustly stains NSCLC, B-cell lymphoma and to a lesser extent breast carcinoma. Furthermore we show for the first time that it is a specific splice variant of CRMP2 that localises to the nucleus of cancer cells. We propose that CDK5 regulation of CRMP2A could contribute to cancer initiation and progression, and this is supported by recent evidence implicating CDK5 activity in taxol-induced cancer metastasis [61]. Phosphorylation of CRMP2 by CDK5 is associated with altered function in neurons [62], however the role of phosphorylation of CRMPs by CDK5 in cancer has not yet been studied. We demonstrate that there are no inherent differences in the activity of p35/CDK5 and p25/CDK5 towards any substrates tested. Whilst the CRMP4 isoform is proposed as a metastasis suppressor in prostate cancer the role of CRMP4 phosphorylation in this action has not been investigated [63]. However our data questions whether CRMP4 is a substrate for CDK5 in healthy cells, or when we increase CDK5 expression. Therefore we propose the CDK5 upregulation would influence CRMP2 but not CRMP4, and furthermore propose that it is the CRMP2A isoform that is a novel oncogenic target for CDK5. This work provides the opportunity for development of additional tools aimed at this CDK5-CRMP2A axis to combat cancer initiation, progression and metastasis.

## Additional files

Additional file 1: Table S1. Peptide Sequences used in Fig. 1. (DOC 30 kb)
Additional file 2: Figure S1. A- GST-Tau was incubated with p35/CDK5 or p25/CDK5 and [γ-32P]-ATP for the times indicated, then subjected to SDS-PAGE prior to autoradiography (upper section). Tau bands were digested with Lys-C and phosphopeptides isolated and identified as described in Methods. A major phosphopeptide eluted with mass/charge ratio of 802.4331 corresponding to the peptide containing Ser235 (lower section), and a second doubly phosphorylated peptide corresponding to a peptide including Ser202 and Thr205 was also identified but at much lower abundance. The same result was obtained from two different phosphorylation reactions. B- GST-tau was phosphorylated as above but using non-radioactive ATP. Proteins were transferred to nitrocellulose after SDS-PAGE and probed with the indicated antibodies. Data is representative of two experiments. **Figure S2.** Co-expression of CDK5 complexes with CRMP2 and CRMP4. Hela cells were co-transfected with equal amounts of expression constructs for CDK5 catalytic subunit, p35 or p25 and FLAG-tagged CRMP2 (A and B) or CRMP4 (C and D) as indicated. Cells were lysed and protein expression and phosphorylation assessed by Western blot analysis (A and C). Quantification was performed on a Licor Odyssey (B and D) with data shown as mean ± S.E.M of three experiments in duplicate. *t*-test, **P* < 0.05, ***P* < 0.01, ****P* < 0.001. **Figure S3.** Co-expression of CDK5 complexes with tau. Hela cells were co-transfected with equal amounts of expression constructs for CDK5 catalytic subunit, p35 or p25 and tau. Cells were lysed and protein expression and phosphorylation assessed by (A) Western blot analysis. (B-D) Quantification was performed on a Licor Odyssey and the ratio between phospho-tau: total tau calculated for each phospho-tau antibody. Data shown as mean ± S.E.M. for three experiments performed in duplicate. *t*-test, **P* < 0.05, ***P* < 0.01, ****P* < 0.001. (PDF 2557 kb)
