# Supplementary material for: Phosphorylation of a splice variant of collapsin response mediator protein 2 in the nucleus of tumour cells links cyclin dependent kinase-5 to oncogenesis
Source: BMC Cancer. 2015 Nov 10;15:885. doi: 10.1186/s12885-015-1691-1 (PMC4640224; doi:10.1186/s12885-015-1691-1)
Supplement: Additional file 1: Table S1. — Peptide Sequences used in Fig. 1. (DOC 30 kb) [file 12885_2015_1691_MOESM1_ESM.doc]

Supplementary Table 1- Peptide Sequences used in Figure 1.

| Peptide Sequence | Name | Figure |
| --- | --- | --- |
| PKTPKKRKKL | TPKKR | 1A |
| PKTPKAAKKL | TPKAA | 1A |
| KKASAPKSPRKPRS | Class 1 (1.0) | 1B, 1C and 1D |
| KKASAPVSPPRDRK | Class 2 | 1B |
| KKPKSRRSPPSIPT | Class 3 | 1B |
| KKENNVLSPLPSQA | Class 4 | 1B |
| KKASAPKSPARPRS | Class 1 (1.1) | 1C and 1D |
| KKASAPVSPRKPRS | Class 1 (1.2) | 1C and 1D |
| KKASAPKSPPKPRS | Class 1 (1.3) | 1C and 1D |
| KKASAAKSPPKPRS | Class 1 (1.4) | 1C |
