# Supplementary material for: Phosphorylation of a splice variant of collapsin response mediator protein 2 in the nucleus of tumour cells links cyclin dependent kinase-5 to oncogenesis
Source: BMC Cancer. 2015 Nov 10;15:885. doi: 10.1186/s12885-015-1691-1 (PMC4640224; doi:10.1186/s12885-015-1691-1)
Supplement: Additional file 2: Figure S1. — A- GST-Tau was incubated with p35/CDK5 or p25/CDK5 and [γ-32P]-ATP for the times indicated, then subjected to SDS-PAGE prior to autoradiography (upper section). Tau bands were digested with Lys-C and phosphopeptides isolated and identified as described in Methods. A major phosphopeptide eluted with mass/charge ratio of 802.4331 corresponding to the peptide containing Ser235 (lower section), and a second doubly phosphorylated peptide corresponding to a peptide including Ser202 and Thr205 was also identified but at much lower abundance. The same result was obtained from two different phosphorylation reactions. B- GST-tau was phosphorylated as above but using non-radioactive ATP. Proteins were transferred to nitrocellulose after SDS-PAGE and probed with the indicated antibodies. Data is representative of two experiments. Figure S2. Co-expression of CDK5 complexes with CRMP2 and CRMP4. Hela cells were co-transfected with equal amounts of expression constructs for CDK5 catalytic subunit, p35 or p25 and FLAG-tagged CRMP2 (A and B) or CRMP4 (C and D) as indicated. Cells were lysed and protein expression and phosphorylation assessed by Western blot analysis (A and C). Quantification was performed on a Licor Odyssey (B and D) with data shown as mean ± S.E.M of three experiments in duplicate. t-test, *P < 0.05, **P < 0.01, ***P < 0.001. Figure S3. Co-expression of CDK5 complexes with tau. Hela cells were co-transfected with equal amounts of expression constructs for CDK5 catalytic subunit, p35 or p25 and tau. Cells were lysed and protein expression and phosphorylation assessed by (A) Western blot analysis. (B-D) Quantification was performed on a Licor Odyssey and the ratio between phospho-tau: total tau calculated for each phospho-tau antibody. Data shown as mean ± S.E.M. for three experiments performed in duplicate. t-test, *P < 0.05, **P < 0.01, ***P < 0.001. (PDF 2557 kb) [file 12885_2015_1691_MOESM2_ESM.pdf]

Supp Fig.1A

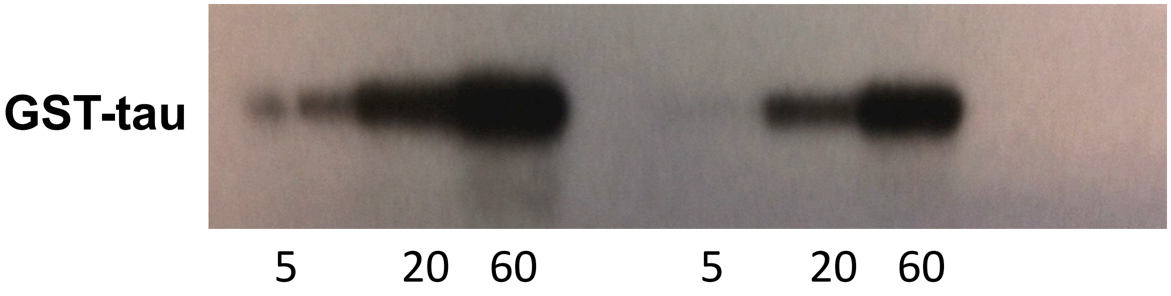

p35/Cdk5 **p25/Cdk5**

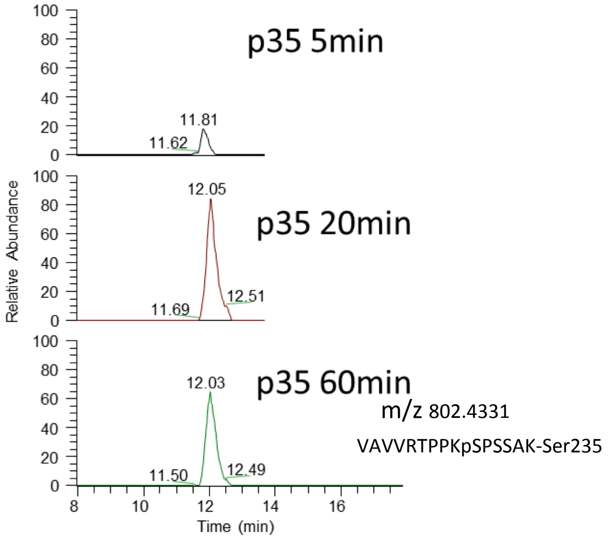

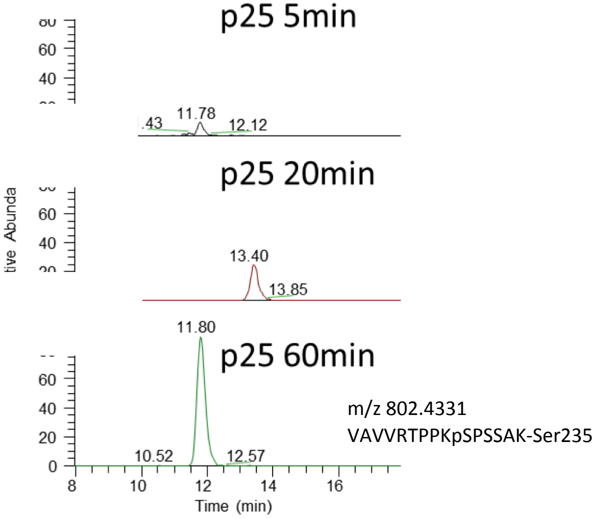

Supp Fig.1B

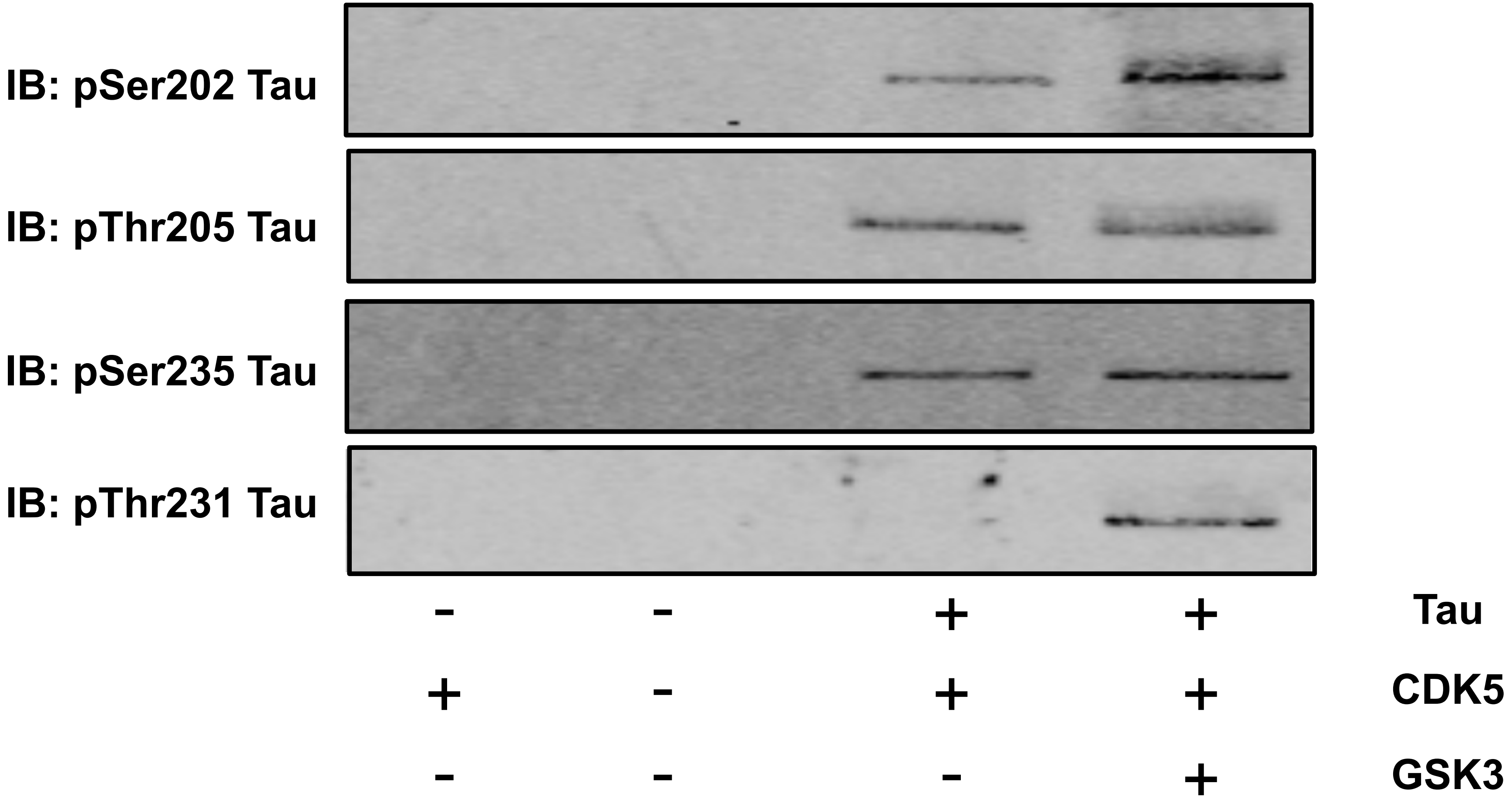
IB: pSer202 Tau
IB: pThr205 Tau
IB: pSer235 Tau
IB: pThr231 Tau
- - + + Tau
+ - + + CDK5
- - - + GSK3

Supp Fig.2

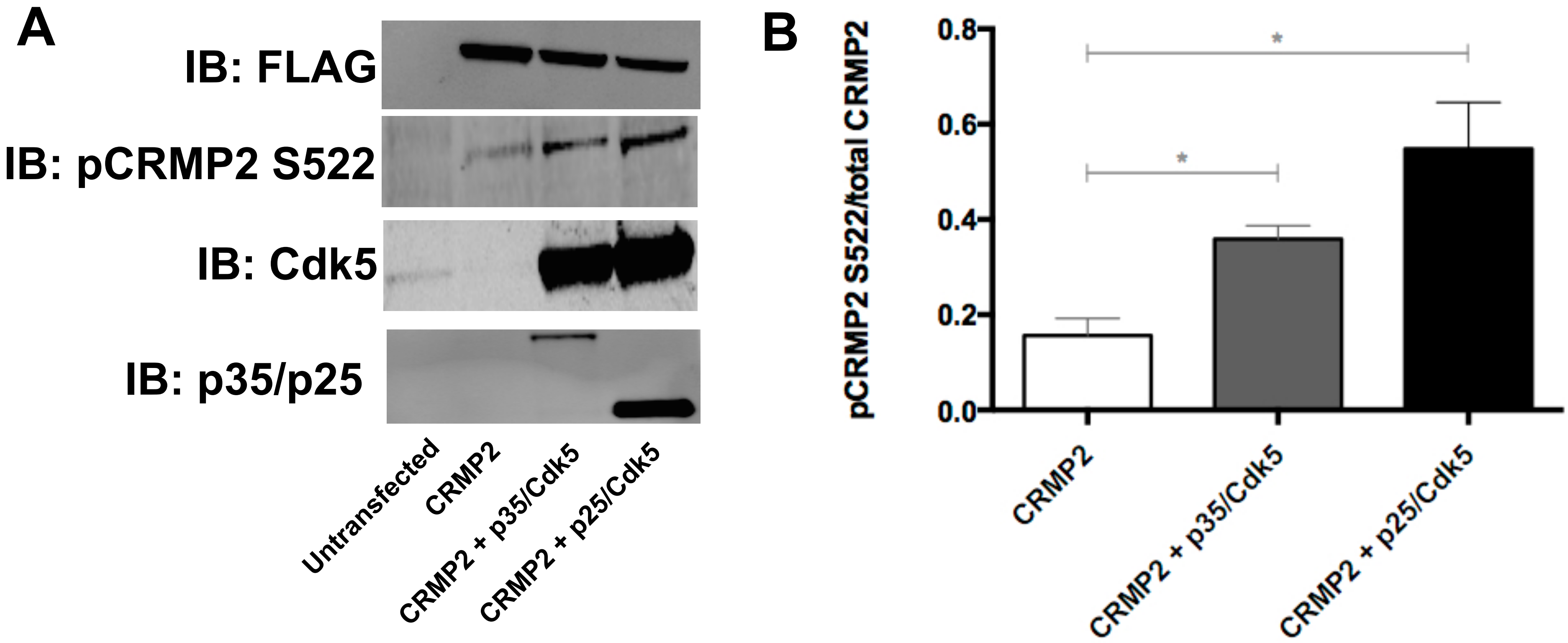
A
IB: FLAG
IB: pCRMP2 S522
IB: Cdk5
IB: p35/p25
Untransfected
CRMP2
CRMP2 + p35/Cdk5
CRMP2 + p25/Cdk5
B
pCRMP2 S522/total CRMP2
0.8
0.6
0.4
0.2
0.0
*
*
CRMP2
CRMP2 + p35/Cdk5
CRMP2 + p25/Cdk5

Supp Fig.2

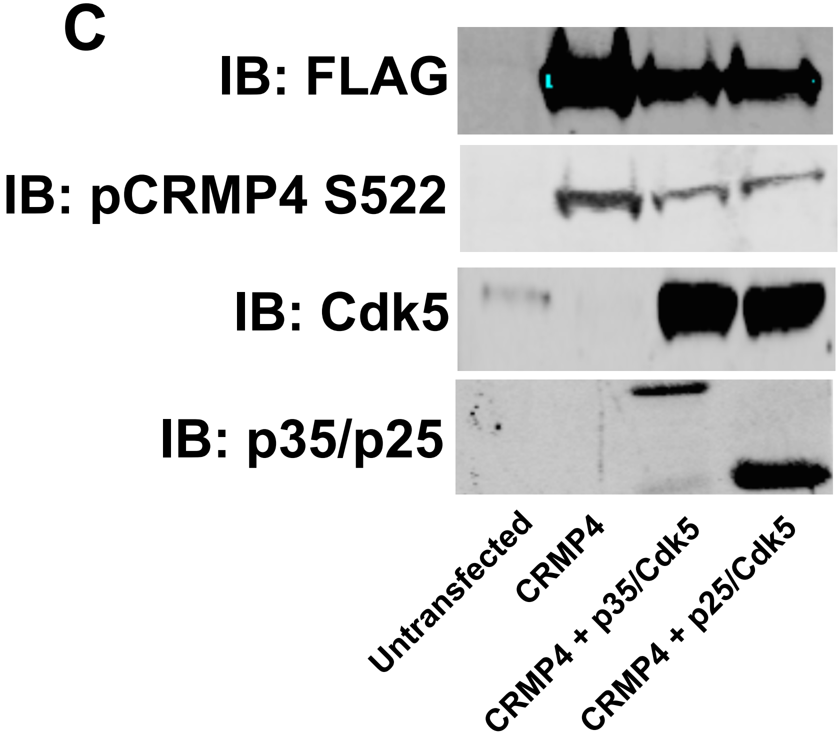
C
IB: FLAG
IB: pCRMP4 S522
IB: Cdk5
IB: p35/p25
Untransfected
CRMP4
CRMP4 + p35/Cdk5
CRMP4 + p25/Cdk5

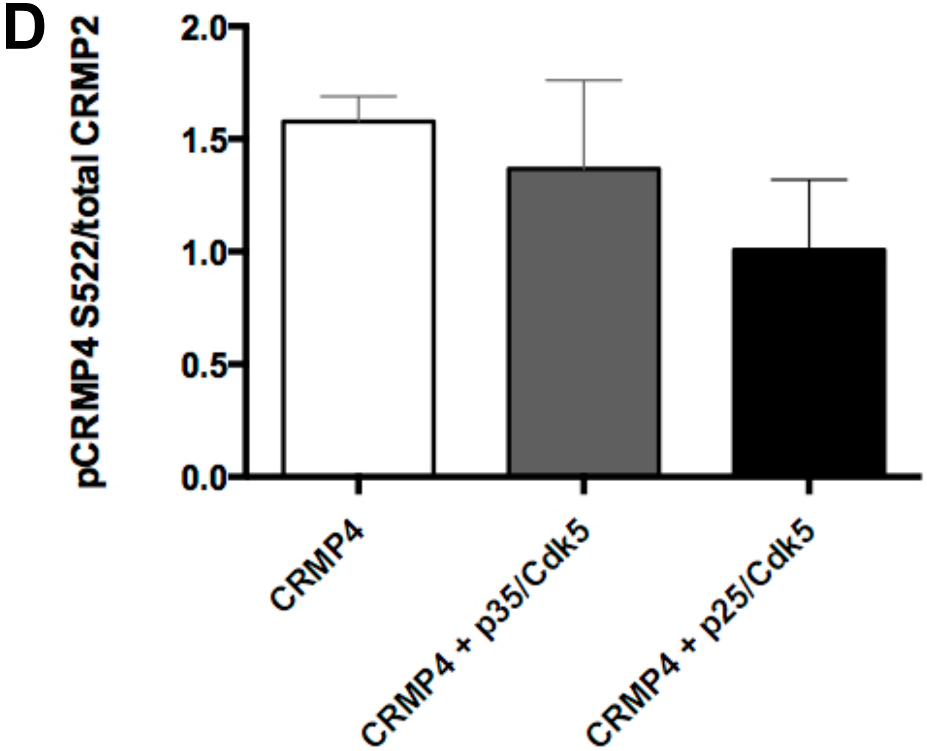
D
pCRMP4 S522/total CRMP2
2.0
1.5
1.0
0.5
0.0
CRMP4
CRMP4 + p35/Cdk5
CRMP4 + p25/Cdk5

Supp Fig.3

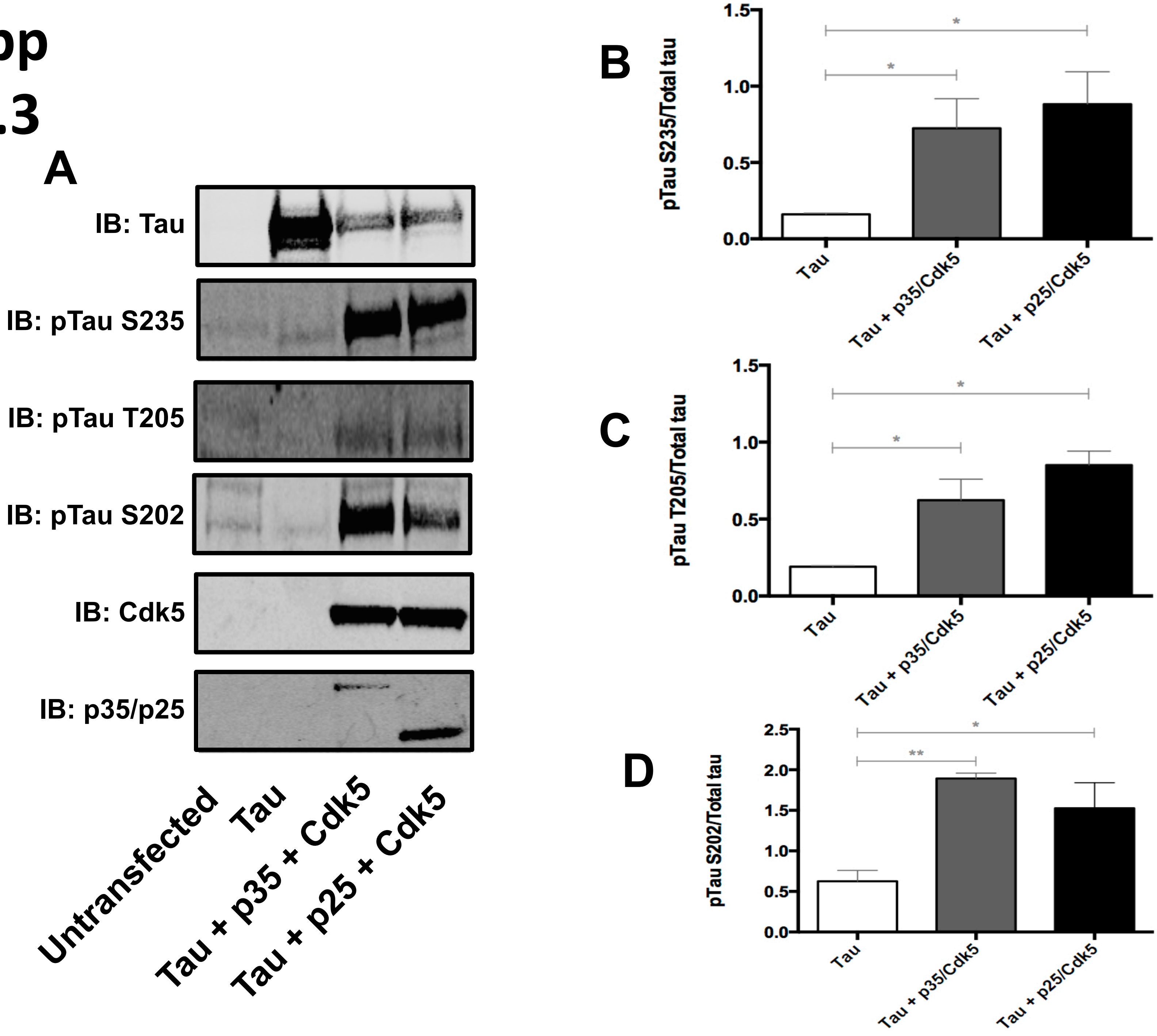
A
IB: Tau
IB: pTau S235
IB: pTau T205
IB: pTau S202
IB: Cdk5
IB: p35/p25
Untransfected
Tau
Tau + p35 + Cdk5
Tau + p25 + Cdk5
B
pTau S235/Total tau
1.5
1.0
0.5
0.0
*
*
Tau
Tau + p35/Cdk5
Tau + p25/Cdk5
C
pTau T205/Total tau
1.5
1.0
0.5
0.0
*
*
Tau
Tau + p35/Cdk5
Tau + p25/Cdk5
D
pTau S202/Total tau
2.5
2.0
1.5
1.0
0.5
0.0
*
**
Tau
Tau + p35/Cdk5
Tau + p25/Cdk5
